# Draft Genome Sequence of Vancomycin-Susceptible, Ampicillin-Intermediate *Enterococcus faecium* Strain D344RRF

**DOI:** 10.1128/genomeA.01720-15

**Published:** 2016-05-05

**Authors:** Mónica García-Solache, Louis B. Rice

**Affiliations:** Department of Medicine, Rhode Island Hospital, Warren Alpert Medical School of Brown University, Providence, Rhode Island, USA

## Abstract

*Enterococcus faecium* is an important nosocomial pathogen, causing a substantial health burden due to high resistance to antibiotics and its ability to colonize the gastrointestinal tract. Here, we present the draft genome of vancomycin-susceptible, ampicillin-intermediate strain D344RRF, a rifampicin/fusidic acid-resistant and commonly used laboratory strain, which is useful in studying the transfer of antibiotic resistance.

## GENOME ANNOUNCEMENT

*Enterococcus faecium* is an increasingly important pathogen in hospitalized patients (1). *E. faecium* presents a particular challenge as a nosocomial infection due to high intrinsic levels of antibiotic resistance and a remarkable capacity to acquire resistance to most clinically relevant antibiotics. Mobile genetic elements are of fundamental importance in the transfer and acquisition of antibiotic resistance determinants by enterococci (2). In this context, strains that are able to acquire resistance determinants and that have useful markers for selection, like those of D344RRF, are a very useful tool to study the mechanisms of transfer, mobilization, and acquisition of DNA between strains, improving our knowledge in this difficult-to-treat pathogen.

Total DNA from *E. faecium* D344RRF was extracted with the Qiagen Genomic tip-100 (Qiagen, Valencia, CA). A DNA library was generated using the Nextera XT DNA sample preparation kit and Nextera XT index primers (Illumina, San Diego, CA). Sequencing was performed on an Illumina MiSeq version 2 instrument as 2 × 150-bp paired-end reads. *De novo* assembly was done using Genomics Workbench version 6.5 (CLC bio, Cambridge, MA). Fastq files were trimmed for quality, minimum length of 50 bp and assembled at high stringency (length fraction, 0.9; similarity fraction, 0.99) using default mismatch/insertion/deletion costs. The contig order and gene synteny were analyzed by comparing the D344RRF draft genome versus the fully closed genomes of *E. faecium* DO and *E. faecium* Au0004 (accession no. ASM17439v2 and GCA_000250945.1) with Mauve 2.3.1 (3). The genome was annotated by the NCBI Prokaryotic Genome Annotation Pipeline. The draft genome sequence of D344RRF is 2,982,307 bp, distributed in 207 contigs, with a G+C content of 37.9% and 2,847 protein-coding genes.

### Nucleotide sequence accession numbers.

This whole-genome shotgun project has been deposited at DDBJ/EMBL/GenBank under the accession no. LOQQ00000000. The version described in this paper is version LOQQ01000000.
